# Comprehensive analysis of circular RNAs in porcine small intestine epithelial cells associated with susceptibility to *Escherichia coli* F4ac diarrhea

**DOI:** 10.1186/s12864-022-08994-8

**Published:** 2023-04-22

**Authors:** Qingyao Zhao, Qinglei Xu, MA. Serafino, Qin Zhang, Chuduan Wang, Ying Yu

**Affiliations:** 1 Key Laboratory of Animal Genetics, Breeding and Reproduction, Ministry of Agriculture & National Engineering Laboratory for Animal Breeding, College of Animal Science and Technology, China Agricultural University, Beijing, 100193 People’s Republic of China; 2 School of Natural Resources and Environmental Studies, University of Juba, B. O. Pox 82, Juba, South Sudan; 3 College of Animal Science and Veterinary Medicine, Shandong Agricultural University, Shandong, 271018 China

**Keywords:** Diarrhea, ETEC-F4ac, circRNA, Intestine epithelial cells, Susceptibility and resistance

## Abstract

**Background:**

Diarrhea is one of the most common diseases in pig industry, which seriously threatens the health of piglets and causes huge economic losses. Enterotoxigenic Escherichia coli (ETEC) F4 is regarded as the most important cause of diarrhea in piglets. Some pigs are naturally resistant to those diarrheas caused by ETEC-F4, because they have no F4 receptors (F4R) on their small intestine epithelial cells that allow F4 fimbriae adhesion. Circular RNA (circRNA) has been shown to play an important regulatory role in the pathogenesis of disease. We hypothesized that circRNAs may also regulate the adhesion of piglet small intestinal epithelial cells to ETEC F4 fimbriae. However, the circRNA expression profiles of piglets with different Enterotoxigenic Escherichia coli F4 fimbriae (ETEC-F4ac) adhesion phenotypes are still unclear, and the intermediate regulatory mechanisms need to be explored. Hence, the present study assessed the circRNA expression profiling in small intestine epithelial cells of eight male piglets with different ETEC-F4 adhesion phenotypes and *ITGB5* genotypes to unravel their regulatory function in susceptibility to ETEC-F4ac diarrhea. Piglets were divided into two groups: non-adhesive group (*n* = 4) with CC genotype and adhesive group (*n* = 4) with TT genotype.

**Results:**

The RNA-seq data analysis identified 13,199 circRNAs from eight samples, most of which were exon-derived. In the small intestine epithelial cells, 305 were differentially expressed (DE) circRNAs between the adhesive and non-adhesive groups; of which 46 circRNAs were upregulated, and 259 were downregulated. Gene ontology and KEGG enrichment analysis revealed that most significantly enriched DE circRNAs’ host genes were linked to cytoskeletal components, protein phosphorylation, cell adhesion, ion transport and pathways (such as adherens junction, gap junction) associated with ETEC diarrhea. The circRNA-miRNA-mRNA interaction network was also constructed to elucidate their underlying regulatory relationships. Our results identified several candidate circRNAs that affects susceptibility to ETEC diarrhea. Among them, circ-SORBS1 can adsorb ssc-miR-345-3p to regulate the expression of its host gene *SORBS1*, thus improving cell adhesion.

**Conclusion:**

Our results provided insights into the regulation function of circRNAs in susceptibility to ETEC diarrhea of piglets, and enhanced our understanding of the role of circRNAs in regulating ETEC diarrhea, and reveal the great potential of circRNA as a diagnostic marker for susceptibility of ETEC diarrhea in piglets.

**Supplementary Information:**

The online version contains supplementary material available at 10.1186/s12864-022-08994-8.

## Background

Diarrhea is the most common disease in pig industry, which seriously endangers the health of piglets and the stable development of pig industry. Enterotoxigenic Escherichia coli (ETEC) expressing the F4 fimbriae is a major cause of diarrhea in neonatal and pre-weaned piglets [1], which leads to considerable economical loss in the pig industry. Three antigenic variants of F4 have been described: F4ab, F4ac and F4ad, of which F4ac is the most prevalent [2]. Studies have shown that the resistance and susceptibility phenotype of piglets to *E. coli* diarrhea is determined by the presence or absence of F4 receptors on their small intestinal epithelial cells [3]. In our previous study, *ITGB5* was found to be a key gene related to the adhesion phenotypes (Small intestinal epithelial cells with or without F4 receptors) [4]. *ITGB5* genotyping can effectively distinguish piglets susceptible or resistant to *E. coli* diarrhea [5]. The small intestinal epithelial cells of piglets with CC genotype were non-adhesive and resistant to ETEC-F4ac diarrhea, whereas piglets with TT genotype were adhesive and susceptible to ETEC-F4ac diarrhea.

Circular RNA (circRNA), a novel class of non-coding RNA, is characterized by a closed-loop structure generated by pre-mRNA back splicing [6]. Different from the traditional linear RNA (including 5 'and 3' tail), circRNA molecules have a closed ring structure and are not affected by RNA exonuclease, thus their expression is more stable and not easy to degrade [7]. Using high-throughput RNA sequencing (RNA-seq) techniques, recent results have shown that a large number of circRNAs are endogenous, stable and widely expressed in mammalian cells, often exhibiting cell type-specific, tissue-specific or developmental stage-specific expression [8–10]. In terms of function, recent studies have shown that circRNA molecules are rich in microRNA (miRNA) binding sites and act as miRNA sponges in cells [11], thereby relieving the inhibition of miRNA on target genes and increasing the expression level of target genes. This mechanism of action is known as the competitive endogenous RNA (ceRNA) mechanism. Li et al. [12] found that a novel circ-PPARA could promote the formation of intramuscular fat in pigs through the adsorption of miR-429 and miR-200b. In addition, circRNA are involved in the development of various disease conditions, such as cardiovascular diseases [13], diabetes [14], neurological diseases [15] and cancer [16]. Emerging evidence suggests that circRNAs may be potential new clinical diagnostic markers or therapeutic approaches for many diseases [17]. Yan et al. [18] investigated circRNA expression profiles in spleen of piglets infected with Clostridium perfringens type C, identifying eight circRNAs associated with necrotizing enteritis caused by Clostridium perfringens type C. Chen et al. [19] analyzed the circRNA expression profile during porcine endemic diarrhea virus (PEDV) infection in IPEC-J2 cell line and identified 26,670 candidate circRNAs.

In this study, we comprehensively analyzed characteristics of circRNA in the adhesive and non-adhesive small intestinal epithelial cells of piglets using RNA-seq data and bioinformatics methods, and explored the role of DE circRNAs in susceptibility to ETEC-F4ac. We also constructed ceRNA network including circRNA, miRNA and mRNA to identify molecule markers involved in susceptibility and resistance to ETEC-F4ac Diarrhea. The results of this study enhance our understanding of the role of circRNAs in regulating ETEC diarrhea resistance, and reveal the great potential of circRNA as a therapeutic target to biological treatment for ETEC diarrhea in piglets.

## Results

### Characterization of circRNAs in porcine small intestinal epithelial cells

**Fig. 1:**
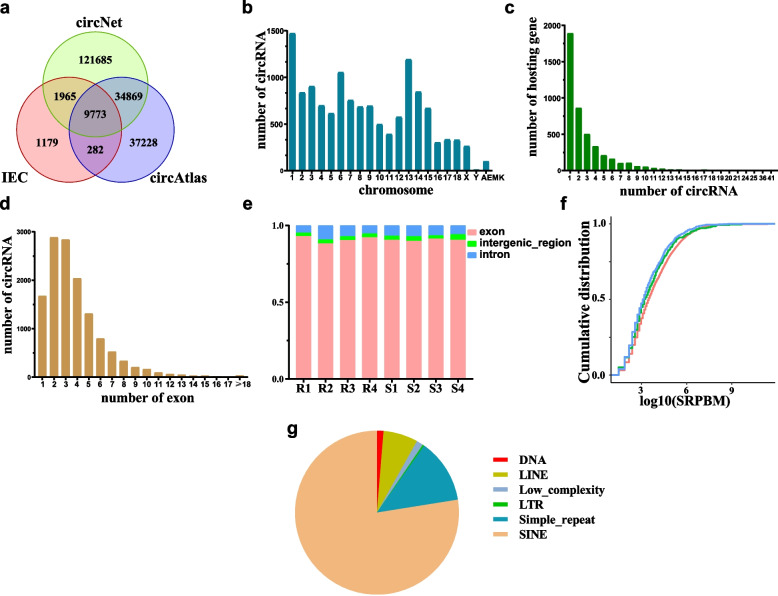
Profiling of circRNA in small intestinal epithelial cells of piglets. **a** CircRNAs identified in piglet intestinal epithelial cells (IEC) were overlapped with circRNAs annotated in circNet and circAtlas databases. **b** The distribution of identified circRNAs in different chromosomes. **c** The distribution of host genes encoding different number of circRNAs in piglet intestinal epithelial cells. **d** The distribution of number of exons that form the exonic circRNAs. **e** The proportion of different categories of circRNAs in intestinal epithelial cells of piglets in each sample. **f** Cumulative distribution of different categories of circRNA expression. **g** The proportion of different categories of RCMs in the flanking introns of exonic circRNAs. IEC: intestinal epithelial cells; SRPBM: spliced reads per billion mapping; LINE: long interspersed nuclear elements; LTR: long terminal repeat; SINE: short interspersed nuclear element

A summary of RNA-seq data from eight porcine small intestinal epithelial cell samples is shown in Table 1. Two kinds of software (find_circ and CIRI2) were used for identification of circRNAs based on back-spliced reads produced from high-throughput RNA sequencing data. We identified 13,199 circRNAs with at least two independent back-spliced junction reads via two kinds of software (Additional file 1: Table S1). The obtained 13,199 circRNAs were aligned with circNet [20] and circAtlas [21] databases, among which 12,020 circRNAs (91%) were known, and 1179 circRNAs were novel (Fig. 1a). Chromosome distribution of those circRNAs is concentrated on chromosome 1, and the least on chromosome Y. Some circRNAs come from mitochondria, and the rest of the chromosomes are roughly evenly distributed (Fig. 1b). Most of the circRNAs identified were exonic circRNA (12,079, accounting for about 91.5%), and 796 circRNAs were intronic (Fig. 1e). These circRNAs were generated from 4400 genes, among which 42.95% genes produced only one circRNA isoform (Fig. 1c). The remaining 324 circRNAs originated from intergenic regions. The most circRNAs were made up of 2–3 exons, and few are more than 10 exons (Fig. 1d). The counts of the back-spliced junction reads of 8 samples were normalized as the spliced reads per billion mapping (SRPBM) (Additional file 1: Table S2). The circRNAs originated from exon, intron and intergenic region showed no significant changes in expression abundance (SRPBM) (Fig. 1f). Then we aligned the flanking intron pairs of exonic circRNAs to identify reverse complementary matches (RCMs) using Basic Local Alignment Search Tool (BLAST). Among the identified RCMs, Short interspersed nuclear element (SINE) and Simple_repeat accounted for 77.5% and 12.7%, respectively (Fig. 1g).

**Table 1:** Summary of RNA-seq data

group	sample ID	raw reads	clean reads	mapping rate
non-adhesive group	R1	123,574,890	120,545,958	92.45%
	R2	115,189,226	113,294,846	92.81%
	R3	127,370,354	125,627,966	95.11%
	R4	122,342,616	120,851,962	95.00%
adhesive group	S1	126,569,978	124,768,650	94.14%
	S2	130,924,082	129,340,552	95.01%
	S3	126,760,832	124,748,322	95.65%
	S4	126,165,252	124,240,216	93.87%

### Differentially expression circRNAs in piglet small intestinal epithelial cells that susceptible/resistant to diarrhea

**Fig. 2:**
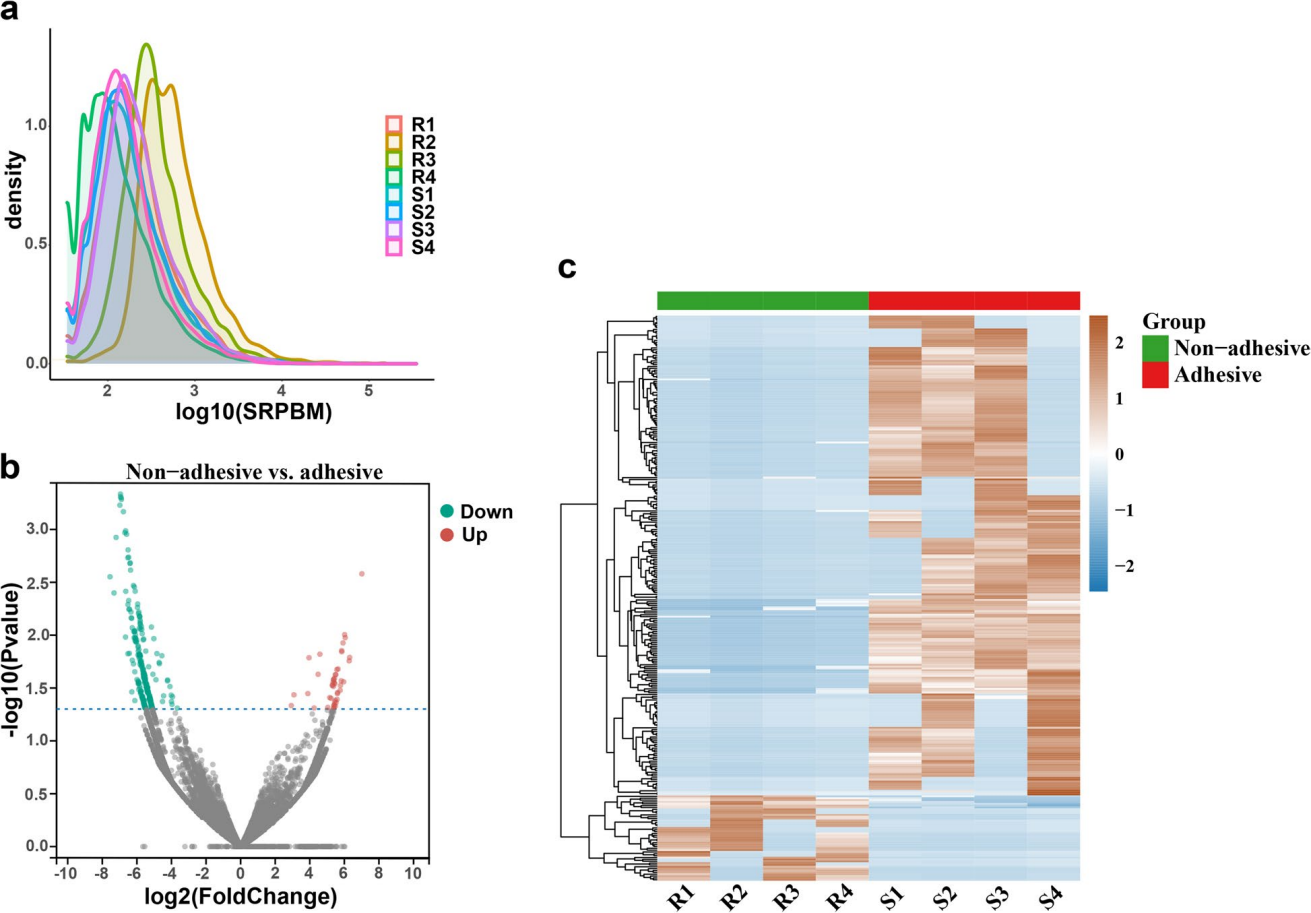
DE circRNAs in porcine small intestine epithelial cell with adhesive and non-adhesive group. **a** Density curves of circRNA expression levels in different samples. **b** Volcano plots depicting DE circRNAs in adhesive group and non-adhesive group. **c** Heatmap showing the expression of all DE circRNAs identified in this research

We further compared the circRNAs in porcine intestinal epithelial cells of the adhesive and non-adhesive groups to identify molecular markers associated with *E. coli* diarrhea. No significant difference was observed in the distribution density of circRNA expression determined based on the SRPBM value obtained from the RNA-seq data of the two groups (Fig. 2a). Next, 305 differentially expressed circRNAs (DEC) were obtained by using DEseq2 R package between adhesive and non-adhesive group (Additional file 1: Table S3), of which 259 were down-regulated and 46 were up-regulated in the non-adhesive group (Fig. 2b). The clustering heatmap comparison showed some circRNAs predominately expressed in adhesive group and some mainly expressed in non-adhesive group (Fig. 2c).

### Functional enrichment analysis of differentially expressed circRNAs revealed their regulatory role in the biogenesis of piglets E. coli diarrhea

**Fig. 3:**
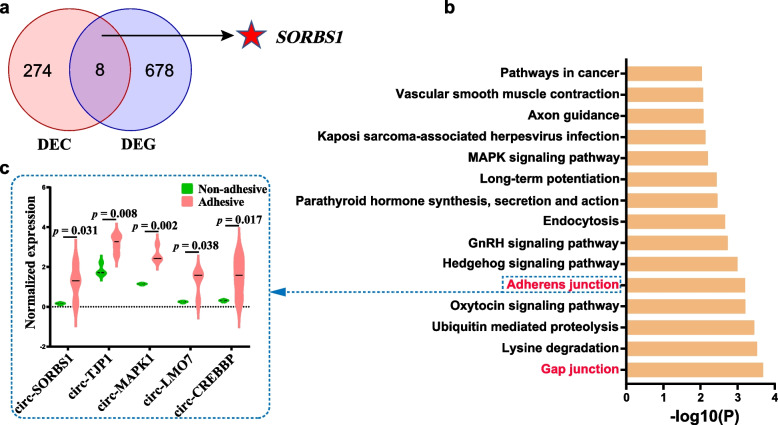
Functional analysis of the DE circRNA host genes. **a** The Venn plot of DE circRNAs’ host genes and DE genes. **b** The top 15 KEGG pathways enriched using host genes of DE circRNAs (*p* < 0.05). **c** Expression levels of circRNAs produced by genes enriched in the adherens junction pathway

The present study performed GO and KEGG analyses of the DE circRNA host genes to elucidate their roles in ETEC-F4ac diarrhea. Functional annotation identified 171 significantly enriched GO terms and 46 significantly enriched pathways (Additional file 1: Table S4). The significantly GO terms of DE circRNA host genes were preferentially enriched in functions related to cytoskeletal components, cell junction, enzyme binding and ion transport. In addition, two pathways related to bacterial adhesion were enriched, namely gap junction and adherens junction (Fig. 3b).

Notably, circRNAs produced by the parental genes involved in the adherens junction pathway were highly expressed in the adhesive group, indicating that these circRNAs may play an important role in regulating the biogenesis of *E. coli* diarrhea (Fig. 3c). Then we performed an overlap analysis of DE circRNA host genes and DE genes between the adhesive group and non-adhesive group, and obtained eight overlapping genes (Fig. 3a), among which sorbin and SH3 domain-containing protein 1 (*SORBS1*) attracted our attention due to it was involved in the adherens junction pathway.

### Construction of a ceRNA network

**Fig. 4:**
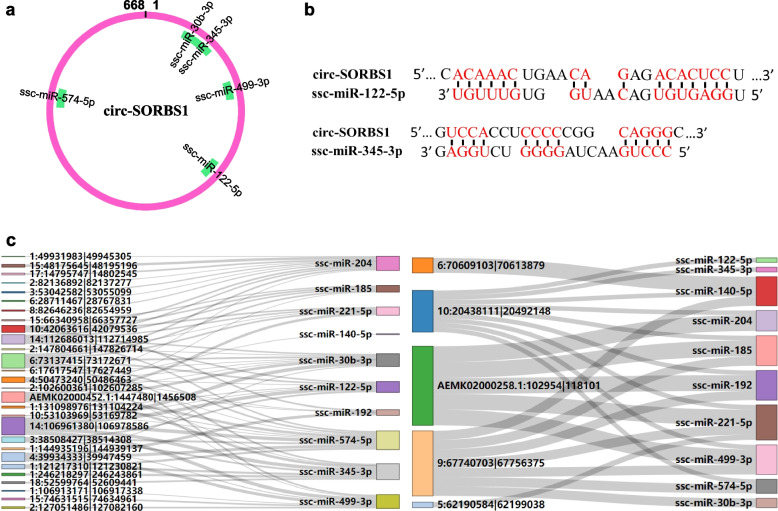
circRNA-miRNA interaction network. **a** miRNA adsorption diagram of circ-SORBS1. **b** The binding sites of circ-SORBS1 with ssc-miR-122-5p and ssc-miR-345-3p. **c** Sanky diagram of downregulated circRNAs (left) and upregulated circRNAs (right) with miRNAs targeting relationship

It was widely accepted that circRNA can act as a miRNA sponge to directly bind to miRNA, and therefore affect the expression levels of the miRNA target genes [22]. Thus, the present study predicted the targeted miRNAs of the DE circRNAs using the miRanda software, so as to explore the potential molecular sponge function of circRNA. The analysis predicted DE circRNAs binding to 451 porcine mature miRNAs and 6485 circRNA − miRNA interactions (Additional file 1: Table S5). Among them, circ-SORBS1 tends to have a lot of miRNA binding sites, indicating its potential molecular sponge function. Figure 4a and b show part of circ-SORBS1’s miRNA binding sites (Fig. 4a, b). Subsequently, 10 miRNAs were selected to construct the circRNA-miRNA interaction network (Fig. 4c), among which 5 were targeted miRNAs of the key candidate circRNA found in this study, and the other 5 selected miRNAs were found to be related to bacterial diarrhea in previous studies [23–27].

**Fig. 5:**
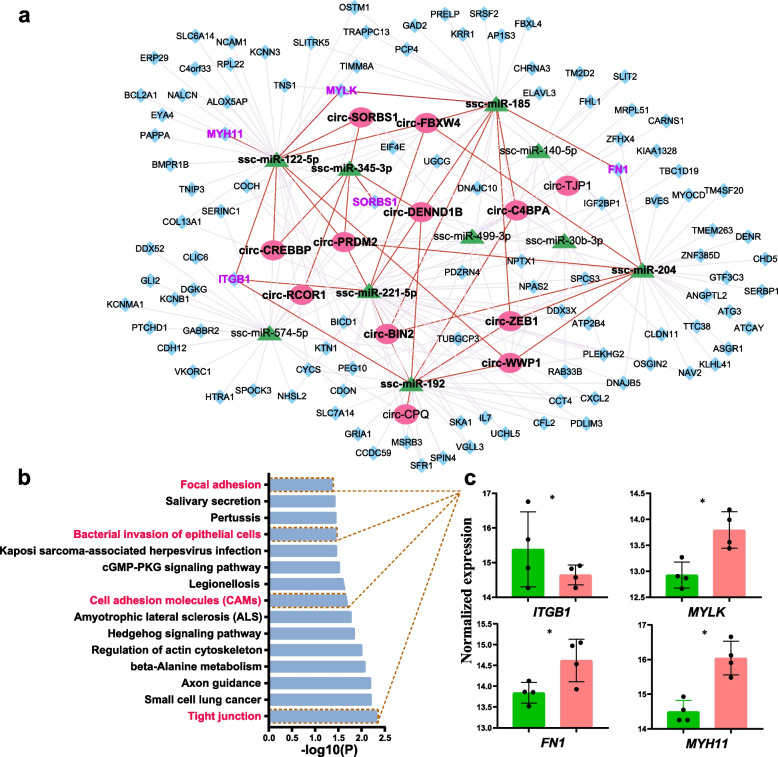
ceRNA network. **a** Interaction network of circRNAs, miRNAs and mRNA. **b** KEGG pathways enriched using the targeted DE genes. **c** Expression levels of genes enriched in pathways associated with *E. coli* diarrhea

Next, we further predicted the targeted genes of these 10 selected miRNAs using TargetScan and miRnet databases, among which 107 were DE genes between the adhesive and non-adhesive groups in the present study. We then performed GO and KEGG analysis on these DE genes (Additional file 1: Table S6). As expected, these DE genes were significantly enriched in four pathways associated with *E. coli* diarrhea, including focal adhesion, bacterial invasion of epithelial cells, cell adhesion molecules and tight junction (Fig. 5b). These pathways mainly include myosin heavy chain 11 (*MYH11*), myosin light chain kinase (*MYLK*), integrin beta-1 (*ITGB1*) and fibronectin (*FN1*) (Fig. 5c), among which *FN1* exists in all four pathways, and *MYH11* is the most significant differentially expressed gene identified in the mRNA data of this study. In addition, *MYLK* have been shown to play an important role in adhesion phenotype in previous study [1].

Finally, we constructed a circRNA-miRNA-mRNA interaction network using Cystoscope software, which contained 12 circRNAs found in this study, 10 predicted miRNA and 107 DE genes (Fig. 5a). In the network, there were 3 upregulated circRNAs (circ-BIN2, circ-DENND1B, circ-C4BPA) in the non-adhesive group, which regulate target gene *ITGB1* by adsorbing ssc-miR-221-5p and ssc-miR-192. While a few downregulated circRNAs, such as circ-CREBBP, circ-WWP1, act as a sponge for ssc-miR-122-5p, ssc-miR-204, probably influencing *MYH11*, *MYLK* and *FN1* target genes. Notably, circ-SORBS1 can regulate the expression of itself host gene *SORBS1* by targeting ssc-miR-345-3p, also can regulate other *E. coli* diarrhea-related genes by targeting ssc-miR-122-5p.

## Discussion

In view of the circRNAs may be involved in the occurrence of disease or can be used as diagnostic markers, we investigated the circRNAs expression profile in small intestinal epithelial cells of Large White piglets resistant or susceptible to ETEC-F4ac diarrhea, and to analysis the function of circRNAs in ETEC-F4ac diarrhea biogenesis. In the present study, we identified 13,199 circRNAs in 8 small intestinal epithelial cells, among which 1179 were not retrievable in the existing circRNA database, indicating that circRNAs in animal tissues have not been fully explored to a large extent.

Previous studies have shown that the resistance and susceptibility phenotype of piglets to *E. coli* diarrhea is determined by the presence or absence of F4 receptors on their small intestinal epithelial cells [3]. In short, the small intestinal epithelial cells of piglets with F4 receptors can adhere to *E. coli* fimbriae and thus showing susceptibility to *E. coli* diarrhea, which is called adhesive phenotype in this study. By contrast, the small intestinal epithelial cells of piglets without F4 receptors exhibit resistance to *E. coli* diarrhea, are referred to as non-adhesive phenotypes. Therefore, in order to reveal the biological function of circRNAs in the adhesion phenotype, we compared the circRNAs expression levels in the adhesive and non-adhesive groups of piglet intestinal epithelial cells and obtained 305 DE circRNAs, of which 46 were up-regulated and 259 down-regulated in the non-adhesive group.

Since circRNAs can function in gene regulation by competing with linear splicing [28], we performed functional enrichment analysis on the host genes of DE circRNAs. The results showed that significantly GO terms of DE circRNA host genes were preferentially enriched in functions related to cytoskeletal components, cell junction, enzyme binding and ion transport. The intestine is composed of a single layer of epithelial cells, which are interconnected by transmembrane proteins and together with the cytoskeleton form a selectively semi-permeable barrier [29, 30], which plays an important role in intestinal homeostasis [31]. Obviously, significant enriched GO term suggests that host genes of DE circRNAs may function by affecting intestinal epithelial cell integrity and permeability, which may be directly related to enterotoxin production by ETEC [32]. Enterotoxins can alter transmembrane protein activity to impair intestinal barrier function, leading to disorder of ion transport, which allows water and electrolytes to enter the intestinal cavity and cause diarrhea [33]. Similarly, there are many other GO terms related to protein phosphorylation. Previous investigations concerning the interactions of ETEC and host eukaryotic cells have shown that ETEC adherence can induces several biochemical changes in target cells, such as inositol phosphate metabolism [34] and activation of protein kinases [35]. As a consequence of the latter, some host cells are phosphorylated. Phosphorylation has also been shown to be associated with the contraction of cytoskeletal protein loops underlying intercellular tight junctions [36], thereby affecting intestinal barrier function. Next, we will also conduct in depth research on phosphorylation phenotype.

In addition, DE circRNAs were also enriched in two KEGG pathways (gap junction and adherens junction) related to bacterial adhesion, which are the main forms of interconnection between intestinal epithelial cells [37]. When *E. coli* colonizes the gut, it adheres to the surface of enterocytes, causing the microvilli of infected cells to collapse. The enterocyte alteration results in the release of large amounts of small molecules from host cells, which can lead to diarrhea, and one possible way for small intestinal cells to release small molecules is through the functional gap junction hemichannels [38]. The adherens junction, along with the desmosomes, provide strong adhesive bonds between the intestine epithelial cells and also aids in intracellular communication [39], which may facilitate adhesion and colonization of ETEC on the small intestine epithelial cells, but not by affecting paracellular permeability. We also found that the expression levels of DE circRNAs (circ-SORBS1, circ-TJP1, circ-MAPK1, circ-CREBBP, circ-LMO7) significantly enriched in the adherens junction pathway were higher in the adhesive group than in the non-adhesive group, indicating that this pathway is up-regulated in the adhesive group, which was consistent with the adhesion phenotype susceptibility to *E. coli* diarrhea. Thus, these five circRNAs may be involved in the regulation of adhesion phenotype, among which the host gene *SORBS1* of circ-SORBS1 is also a differentially expressed gene, which has aroused our attention. *SORBS1* gene is required for insulin-stimulated glucose transport. Many studies have found that *SORBS1* gene is closely related to various metabolic diseases such as diabetes [40], obesity [41] and gastrointestinal cancer [42]. In addition, it's involved in formation of actin stress fibers and focal adhesions. suggesting that it plays an important role in the adhesion phenotype. Jin et al. [43] found that *SORBS1* can be targeted by miRNA to regulate the progression of gastric cancer, suggesting that circ-*SORBS1* may regulate the expression of its host genes by targeting miRNA, which was confirmed in the analysis of this study. We found that circ-SORBS1 has the most miRNA binding sites, demonstrating its great potential as a sponge for miRNA molecules. Combined with the above functions of *SORBS1* gene, it is suggested that circ-SORBS1 may be the most potential key circRNA to regulate the adhesion of ETEC to piglet small intestinal epithelial cells.

CircRNAs could negatively regulate the activity of miRNAs as miRNA sponges by competing endogenous RNA (ceRNA) network [11]. In the present study, combining our previous mRNA data and predicted miRNAs, we constructed a circRNA-miRNA-mRNA interaction network that contained 12 circRNAs found in this study, 10 predicted miRNA and 107 DE genes (Fig. 5a). Subsequently, through functional enrichment analysis of DE genes targeted by predicted miRNA, it was found that genes were enriched in pathways closely related to *E. coli* diarrhea, such as tight junction, cell adhesion molecules, bacteria invasion of epithelial cells and focal adhesion, thus we obtained four important candidate genes (*MYLK, ITGB1, MYH11, FN1*) associated with diarrhea. Ren et al. [1] found that *MYLK* is located on SSC13 locus near to a 2.3 Mb region for F4acR, which strongly support that it might play an important role in regulating the adhesion phenotype of small intestinal epithelial cells. In this study, intestinal epithelial cells of piglets were divided into the adhesive group and non-adhesive group based on *ITGB5* genotype and adhesion test. Since *ITGB1* and *ITGB5* belong to the same gene family (integrin family), we speculated that *ITGB1* may play an important role in bacterial adhesion. The expression of invasin protein in E. coli can promote bacterial invasion of host cells [44], and *ITGB1* functions as a receptor for invasin in the bacterial infection pathway [45], which confirming our viewpoint. *FN1*, as a fibronectin, plays a key role in intercellular adhesion, and is beneficial to ETEC colonization [46]. In addition, *MYH11*, as top DE gene, was enriched in tight junction pathway, which acts as an intestinal barrier in *E. coli* infection [37]. Combining the functions of these important candidate genes, we obtained some key targeted regulatory relationships.

In ceRNA network, we found that two downregulated circRNAs (circ-FBXW4, circ-ZEB1) in the non-adhesive group could bind with ssc-miR-185 and target to regulate *MYLK* and *FN1*. Wang et al. [47] found that ssc-miR-185 plays an important regulatory role in piglet diarrhea. Besides that, ssc-miR-185 was associated with various cancers, covering gastric cancer [48], colorectal cancer [49] and pancreatic cancer [50]. Ssc-miR-185 can also play a regulatory role in response the immune inflammatory [51]. Among these miRNAs, ssc-miR-122-5p can be targeted by five downregulated circRNAs (circ-SORBS1, circ-FBXW4, circ-WWP1, circ-PRDM2, circ-CREBBP) and regulate the expression of *MYH11* and *MYLK*, which may be a key miRNA in regulating adhesion phenotype. MiR-122-5p was found to be a biomarker for gastric cancer [52], and can inhibit the proliferation of various cancer cells by targeting downstream genes [53, 54]. Notably, since *ITGB1* was the only upregulated gene in the non-adhesive group, so there were 3 upregulated circRNAs (circ-BIN2, circ-DENND1B, circ-C4BPA) in the non-adhesive group might regulate target gene *ITGB1* by adsorbing ssc-miR-221-5p and ssc-miR-192. Sun et al. [24] found that knocking out ssc-miR-192 enhanced the adhesion of ETEC-F18, which is consistent with the results of this study. In addition, we also found that circ-SORBS1 can regulate the expression of its host gene *SORBS1* by targeting ssc-miR-345-3p. Many studies have shown that miR-345-3p is closely related to inflammation [55, 56], while target gene *SORBS1* plays a role in tyrosine phosphorylation and is involved in formation of actin stress fibers and focal adhesion. Enterotoxin produced by ETEC can disrupt the barrier function of epithelial cells, leading to abnormal electrolyte and water secretion, while triggering mild tissue inflammation [57]. Phosphorylation has also been shown to be associated with the contraction of cytoskeletal protein loops underlying intercellular tight junctions [36], thereby affecting intestinal barrier function.

## Conclusion

In conclusion, we investigated the characteristics of circRNAs in small intestine epithelial cells of piglets, and comprehensively analyzed circRNA-miRNA-mRNA regulatory networks. We identified many circRNAs and pathways associated with intestinal barrier function and susceptibility to diarrheal infection caused by ETEC-F4ac. By constructing the ceRNA network, we found many candidate circRNAs and their regulatory mechanisms that may regulate E. coli diarrhea. For example, circ-SORBS1 may regulate the expression of its host gene *SORBS1* by targeting SSC-345-3p, and play a role in the biogenesis of *E. coli* diarrhea. Our study reveals the great potential of circRNA as a diagnostic marker to biological treatment for diarrhea in piglets. On the other hand, circRNA can also be used as a biomarker to identify susceptibility to diarrhea in piglets to support breeding for disease resistance.

## Methods

### Experimental animals and sample collection

All the experiments carried out in this study followed the Animal Welfare Committee’s approved protocol of Agricultural University (Permit Number: DK996). The information of experimental animals was described in our previous study [58]. Briefly, healthy male Large White piglets raised in the experimental farm of the Chinese Academy of Agricultural Sciences were selected as experimental animals. The piglets were humanely euthanized and slaughtered exactly at the weaning age of 35 days. Specifically, Piglets were first humanely euthanized by using Carbon dioxide (CO2) inhalation method prior to slaughter. To make the piglets unconscious in the shortest possible time, the gas chamber (61 cm × 38 cm × 46 cm) with a sealable lid and a gas inlet was pre-filled with CO2, then the piglets in groups of four each were placed into the chamber. Gas was continuously pumped into the chamber, and the piglets became unconscious. To confirm this unconsciousness, palpebral reflex and response to pinprick on the nose were performed every 30 s after the piglet assumed a loss of posture. The piglets were confirmed dead after there were no more palpebral reflexes and breathing. Small intestinal tissue samples were collected aseptically from each animal within 30 min after slaughter. Each tissue sample was cut longitudinally, rinsed with a hypotonic EDTA solution (5 mmol/L EDTA, pH = 7.4), placed in cryovials and immediately transferred into liquid nitrogen containers. The collected samples were then sent to the laboratory for storage in a -80 °C freezer until using for RNA extraction and adhesion assay later. The experiment was conducted in accordance with the protocol approved by the Animal Welfare Committee of China Agricultural University (Permit Number: DK996).

### Cell adhesion assay

Adhesion phenotype of small intestinal epithelial cells of Large White piglets to ETEC F4ac was determined using an in vitro cell adhesion assay. The ETEC-F4ac strain (C83907, O149:K91) was provided by China Institute of Veterinary Drug Control, Beijing, China. The detailed procedures of the adhesion assay have been described in our previous studies [59]. First, the suspension of brush border of epithelial cells of small intestine and ETEC-F4ac suspension were prepared respectively. Then the bacterial suspension and the brush border cell suspension (0.1 ml each) were mixed with 1μL mannose (0.4 mg/mL), put in the incubation for 30 min at room temperature. Afterwards, a drop of the suspension mixture was placed on a glass slide to detect adhesion phenotype by using a phase-contrast microscopy. For a single epithelial cell, when more than 5 bacteria adhered to its brush border, it was judged to be adhesive. For an individual piglet, over 20 epithelial cells from the epithelial cell specimen from it were checked and the piglet was regarded as strongly adhesive when at least 80% of the epithelial cells were judged as adhesive, adhesive when 10% to 80% of the epithelial cells were adhesive, weakly adhesive when less than 10% of the epithelial cells were adhesive, or non-adhesive when no epithelial cells were adhesive. Adhesive piglets were considered susceptible to ETEC F4ac diarrhea, non-adhesive piglets were regarded as resistant to ETEC F4ac diarrhea.

### Genotyping of piglets

To further determine the association between piglet genotype and adhesion phenotype (Small intestinal epithelial cells with or without F4 receptors), we genotyped the piglets in our previous study using integrin subunit beta 5 (*ITGB5*) SNP NC_010455.5 (g.135577826 C > T) [60]. The association between *ITGB5* and ETEC F4ac susceptibility has been identified by GWAS method in our previous study [4].

Combining the results of cell adhesion assay and genotyping, four (4) pigs with adhesive small intestine epithelial cells with TT genotype were selected as the adhesive group, and another four (4) pigs with non-adhesive small intestine epithelial cells with CC genotype were selected as the non-adhesive group. In this study, the adhesive group was considered susceptible to ETEC-F4ac diarrhea, the non-adhesive group was considered resistance to ETEC-F4ac diarrhea.

### RNA isolation and quality assessment

The total RNA was extracted from small intestine tissues of eight (8) selected piglets by Trizol reagent (Invitrogen, Carlsbad, CA) according to the manufacturer's instruction. The kaiaoK5500®Spectrophotometer (Kaiao, Beijing, China) was adopted to monitor RNA purity. The concentrations of isolated RNA were determined using the Nano Drop spectrophotometer. RNA integrity and concentration was assessed using the RNA Nano 6000 Assay Kit of the Bioanalyzer 2100 system (Agilent Technologies, CA, USA). The quality of all the RNA samples were good enough (OD260/280 > 1.90, RNA integrity number > 8.7) to do the sequencing. Then 20 µL of the isolated total RNA from each sample were sent to company (Annoroad Gene Technology Corporation -Beijing) for sequencing.

### Library preparation for RNA sequencing

A total amount of 2 μg RNA per sample was used as an input material for the RNA library preparations. Sequencing libraries were generated using NEBNext® Ultra™ RNA Library Prep Kit for Illumina® (#E7530L, NEB, USA) following the manufacturer's recommendations and index codes were added to attribute sequences to each sample. Briefly, mRNA was purified from total RNA using poly-T oligo-attached magnetic beads. More details about library preparation have been described in our previously published paper [61]. The libraries were sequenced on an Illumina platform (HiSeq Xten) and 150 bp paired-end reads were generated.

### Quality control for raw reads and circRNA identification

Raw reads in fastq format were firstly processed through quality control. Briefly, reads containing adapter, polyN and low-quality bases at high proportion were removed to obtain clean reads. The above steps have already been done by sequencing company. All the downstream analyses were based on the clean reads with high quality. Reference genome and annotation files were downloaded from genome website (version: Sscrofa11.1; GCA_000003025.6).

The circRNAs were detected by two software of CIRI2 [62] and find_circ [63]. The intersecting results of the two tools were considered as candidate circRNAs and used for subsequent analysis. In this study, the spliced reads per billion mapping (SRPBM) method was used to estimate the circRNA expression level. The calculation formula was as follows:

$$\mathrm{SRPBM }= (\mathrm{Back spliced junction reads }/\mathrm{ Total number of mapped reads})\times 109$$

Candidate circRNAs were annotated using circAnno software [64]. To identify reverse complementary matches (RCMs), we aligned two introns’ sequences flanking the same exonic circRNA using Basic Local Alignment Search Tool (BLAST) with ‘blastn’ task, ‘-word_size 7’ and ‘-evalue 20’. Briefly, the upstream and downstream introns of an exonic circRNA were input as query sequences and subjected sequences, respectively. For each intron pair, several alignments were obtained, and the alignment with lowest e-value that passed the threshold was regarded as reverse complementary match (RCM). Then we downloaded the repetitive sequences in pig from UCSC Table Browser (http://genome.ucsc.edu/cgi-bin/hgTables) and compared it with RCM genomic coordinates to discover significant overlaps of RCMs and repeat elements.

### Differential expression and functional enrichment analysis of circRNA

Differential expression (DE) analysis of adhesive and non-adhesive groups was performed using the DESeq2 R package (1.34.0) based on the negative binomial distribution [65]. Candidate circRNAs subject to criteria of *p* value < 0.05 and |log2(FoldChange)|> 1 were assigned as DE circRNAs between non-adhesive group and adhesive group.

Gene ontology (GO) enrichment and KEGG [66] pathway analysis for parental genes of DE circRNAs were conducted by the KOBAS software [67]. GO terms and KEGG pathway with *p* < 0.05 were considered to be significantly enriched.

### CircRNA-miRNA-mRNA network analysis

Combining our previous mRNA data (PRJNA562774, more details can be found in previously published paper [61].) with predicted DE circRNA targeted miRNA data, we constructed a regulatory network of circRNA-miRNA-mRNA to reveal the potential association of ETEC-F4ac adhesion in small intestinal epithelial cells of piglets. All porcine mature miRNAs sequences published in miRbase [68] (http://www.mirbase.org/) were downloaded for further analysis. In details, miRNA binding sites of all DE circRNAs were predicted using miRanda software with free energies of ≤ -20.0 kcal/mol and paring score ≥ 150. Subsequently, target genes of miRNA were predicted using TargetScan (https://www.targetscan.org/vert_80/) and miRnet [69] (https://www.mirnet.ca/) databases. The intersection results of the DE genes that obtained in our mRNA data and predicted target genes were taken as the candidate target genes. Potential circRNA-miRNA-mRNA interactions were established and visualized using Cytoscape 3.9.1 software [70] (http://cytoscape.org/). Gene ontology (GO) enrichment and KEGG pathway analysis for candidate target genes were conducted by the KOBAS software. GO terms and KEGG pathway with *p* < 0.05 were considered to be significantly enriched.

## Supplementary Information

**Additional file 1: Table S1.** Summary of circRNAs identified in eight samples. **Table S2.** The counts of the back-spliced junction read of 8 samples were normalized as the spliced reads per billion mapping (SRPBM). **Table S3.** Differentially expressed circRNAs (DEC) between adhesive and non-adhesive group. **Table S4.** Significantly enriched GO terms of DE circRNA host genes between adhesive and non-adhesive group. **Table S5.** Predicted DE circRNA's miRNA binding sites. **Table S6.** Significantly enriched GO terms and KEGG pathways of DE genes between adhesive and non-adhesive group.

## Data Availability

Raw sequencing data for the eight samples analyzed in this study have been uploaded to the Sequence Read Archive (SRA) in National Center for Biotechnology Information (NCBI), and are available under accession number: PRJNA562774 or (https://www.ncbi.nlm.nih.gov/bioproject/PRJNA562774). Data generated during analysis are included in the manuscript as supplementary files.

## Abbreviations

ETEC: Enterotoxigenic Escherichia coli
F4ab: Fimbriae 4 ab antigen variant
F4ac: Fimbriae 4 ac antigen variant
F4ad: Fimbriae 4 ad antigen variant
*ITGB5*: Integrin beta-5
RNA-seq: RNA-sequencing
DE: Differentially expressed
GO: Gene ontology
KEGG: Kyoto Encyclopedia of Genes and Genomes
*SORBS1*: Sorbin and SH3 domain-containing protein 1
*E.coli*: Escherichia coli
circRNA: Circular RNA
miRNA: MicroRNA
ceRNA: Competitive endogenous RNA
PEDV: Porcine endemic diarrhea virus
IPEC-J2: Intestinal Porcine Epithelial Cell line-J2
SRPBM: Spliced reads per billion mapping
RCMs: Reverse complementary matches
BLAST: Basic Local Alignment Search Tool
SINE: Short interspersed element
LINE: Long interspersed element
IEC: Intestinal epithelial cells
DEC: Differentially expressed circRNA
DEG: Differentially expressed gene
*MYH11*: Myosin heavy chain 11
*MYLK*: Myosin light chain kinase
*ITGB1*: Integrin beta-1
*FN1*: Fibronectin
mRNA: Messenger RNA
